# Platelet inhibitor withdrawal and outcomes after coronary artery surgery: an individual patient data meta-analysis

**DOI:** 10.1093/ejcts/ezae265

**Published:** 2024-07-05

**Authors:** Michael Schoerghuber, Thomas Kuenzer, Fausto Biancari, Magnus Dalén, Emma C Hansson, Anders Jeppsson, Georg Schlachtenberger, Martin Siegemund, Andreas Voetsch, Gudrun Pregartner, Ines Lindenau, Daniel Zimpfer, Andrea Berghold, Elisabeth Mahla, Andreas Zirlik

**Affiliations:** Division of Anaesthesiology and Intensive Care Medicine 2, Medical University of Graz, Graz, Austria; Institute for Medical Informatics, Statistics and Documentation, Medical University of Graz, Graz, Austria; Department of Internal Medicine, South-Karelia Central Hospital, University of Helsinki, Lappeenranta, Finland; Department of Cardiac Surgery, Karolinska University Hospital, Stockholm, Sweden; Department of Molecular Medicine and Surgery, Karolinska Institutet, Stockholm, Sweden; Department of Molecular and Clinical Medicine, Institute of Medicine, Sahlgrenska Academy, University of Gothenburg, Gothenburg, Sweden; Department of Cardiothoracic Surgery, Sahlgrenska University Hospital, Gothenburg, Sweden; Department of Molecular and Clinical Medicine, Institute of Medicine, Sahlgrenska Academy, University of Gothenburg, Gothenburg, Sweden; Department of Cardiothoracic Surgery, Sahlgrenska University Hospital, Gothenburg, Sweden; Department of Cardiothoracic Surgery, University Hospital of Cologne, Cologne, Germany; Intensive Care Medicine, Department of Acute Medicine, University Hospital Basel, Basel, Switzerland; Department of Clinical Research, University of Basel, Basel, Switzerland; Department of Cardiovascular and Endovascular Surgery, Paracelsus Medical University, Salzburg, Austria; Institute for Medical Informatics, Statistics and Documentation, Medical University of Graz, Graz, Austria; Department of Anaesthesiology and Intensive Care Medicine, Hospital Oberwart, Oberwart, Austria; Division of Cardiac Surgery, University Heart Center Graz, Medical University of Graz, Graz, Austria; Institute for Medical Informatics, Statistics and Documentation, Medical University of Graz, Graz, Austria; Division of Anaesthesiology and Intensive Care Medicine 2, Medical University of Graz, Graz, Austria; Division of Cardiology, University Heart Center Graz, Medical University of Graz, Graz, Austria

**Keywords:** P2Y_12_ receptor inhibitors, Coronary artery bypass grafting, Cardiopulmonary bypass, BARC-4 bleeding, Mortality, Postoperative ischaemic events

## Abstract

**OBJECTIVES:**

To evaluate the association between guideline-conforming as compared to shorter than recommended withdrawal period of P2Y_12_ receptor inhibitors prior to isolated on-pump coronary artery bypass grafting (CABG) and the incidence of severe bleeding and ischaemic events. Randomized controlled trials are lacking in this field.

**METHODS:**

We searched PUBMED, Embase and other suitable databases for studies including patients on P2Y_12_ receptor inhibitors undergoing isolated CABG and reporting bleeding and postoperative ischaemic events from 2013 to March 2024. The primary outcome was incidence of Bleeding Academic Research Consortium type 4 (BARC-4) bleeding defined as any of the following: perioperative intracranial bleeding, reoperation for bleeding, transfusion of ≥5 units of red blood cells, chest tube output of ≥2 l. The secondary outcome was postoperative ischaemic events according to the Academic Research Consortium 2 Consensus Document. Patient-level data provided by each observational trial were synthesized into a single dataset and analysed using a 2-stage IPD-MA.

**RESULTS:**

Individual data of 4837 patients from 7 observational studies were synthesized. BARC-4 bleeding, 30-day mortality and postoperative ischaemic events occurred in 20%, 2.6% and 5.2% of patients. After adjusting for EuroSCORE II and cardiopulmonary bypass time, guideline-conforming withdrawal was associated with decreased BARC-4 bleeding risk in patients on clopidogrel [adjusted odds ratio (OR) 0.48; 95% confidence intervals (CI) 0.28–0.81; *P* = 0.006] and a trend towards decreased risk in patients on ticagrelor (adjusted OR 0.48; 95% CI 0.22–1.05; *P* = 0.067). Guideline-conforming withdrawal was not significantly associated with 30-day mortality risk (clopidogrel: adjusted OR 0.70; 95% CI 0.30–1.61; ticagrelor: adjusted OR 0.89; 95% CI 0.37–2.18) but with decreased risk of postoperative ischaemic events in patients on clopidogrel (clopidogrel: adjusted OR 0.50; 95% CI 0.30–0.82; ticagrelor: adjusted OR 0.78; 95% CI 0.45–1.37). BARC-4 bleeding was associated with 30-day mortality risk (adjusted OR 4.76; 95% CI 2.67–8.47; *P* < 0.001).

**CONCLUSIONS:**

Guideline-conforming preoperative withdrawal of ticagrelor and clopidogrel was associated with a 50% reduced BARC-4 bleeding risk when corrected for EuroSCORE II and cardiopulmonary bypass time but was not associated with increased risk of 30-day mortality or postoperative ischaemic events.

## Graphical abstract

**Figure:**
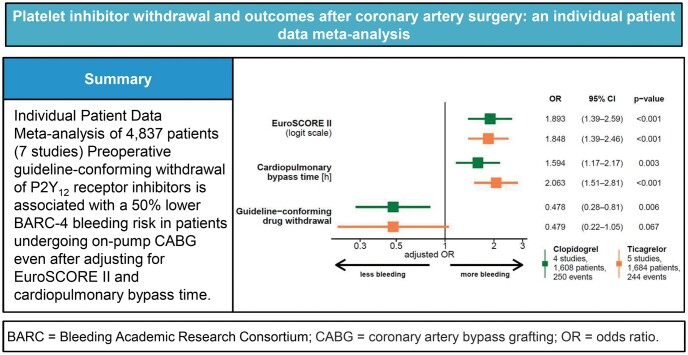

## INTRODUCTION

Dual antiplatelet therapy (DAPT) with aspirin and a P2Y_12_ receptor inhibitor has been the cornerstone for preventing thrombotic complications in patients with acute coronary syndrome (ACS) and after percutaneous coronary intervention, albeit at the expense of increased risk of bleeding [1–3]. To reduce the risk of surgery-related bleeding in patients on P2Y_12_ receptor inhibitors presenting for non-emergent cardiac surgery, current European Society of Cardiology (ESC)/European Association for Cardio-Thoracic Surgery (EACTS) and American Heart Association/American College of Cardiology (AHA/ACC) guidelines have issued a class IIa recommendation for a ‘standardized’ drug-specific preoperative withdrawal period of 3, 5 and 7 days for ticagrelor, clopidogrel and prasugrel, respectively, to allow for the recovery of platelet function [1, 2, 4]. However, there are substantial inter-institutional variations in practice, potential covariates associated with surgery-related bleeding remain unknown and heterogeneous definitions of bleeding have been adopted [5–7]. For relative safety comparisons across studies, Bleeding Academic Research Consortium (BARC) bleeding was introduced in 2011 as a standardized definition for bleeding in patients on antithrombotic therapy but has yet to be generally reported in the literature [8].

In this individual patient data meta-analysis (IPD-MA), we investigated whether guideline-conforming drug-specific preoperative withdrawal as opposed to a shorter than recommended withdrawal period was associated with the incidence of BARC-4 bleeding, 30-day mortality and postoperative ischaemic events according to the Academic Research Consortium 2 Consensus Document [8, 9].

## PATIENTS AND METHODS

### Ethics statement

This IPD-MA performed secondary analyses of existing non-identifiable data and satisfied the criteria for the waiver of ethics review by the local Research Ethics Committee. All studies included in this IPD-MA received approval according to local requirements.

The protocol for this IPD-MA was registered in PROSPERO (CRD42022291946). This review was reported according to the Preferred Reporting Items for Systematic Review and Meta-Analyses of Individual Participant Data (PRISMA-IPD) Statement [10, 11].

### Eligibility and search strategy

**Figure 1:**
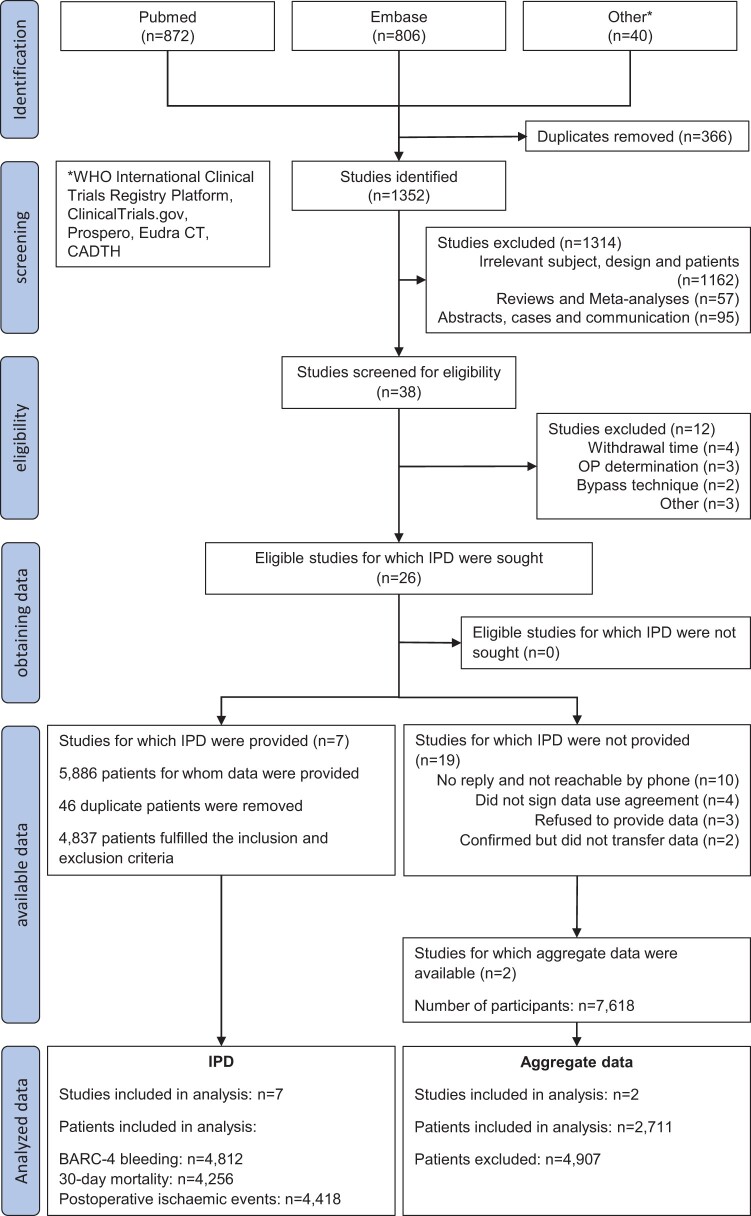
PRISMA 2020 flow diagram for systematic reviews, which included searches of databases and registers investigating bleeding according to type of P2Y_12_ receptor inhibitor and preoperative withdrawal period from July 2013 to March 2024. Studies published until June 2013 have been included in a prior pooled meta-analysis [13].

We searched PUBMED (starting July 2013), Embase (starting January 2014) and other suitable databases (WHO International Clinical Trials Registry Platform, ClinicalTrials.gov, Prospero, Eudra CT and CADTH) until March 2024 for studies including patients on P2Y_12_ receptor inhibitors undergoing isolated coronary artery bypass grafting (CABG) and reporting bleeding and postoperative ischaemic events. Details regarding the rationale and methods have been previously published [12]. The search strategy is presented in Supplementary Material, Table S1, while the flow diagram of the selection process is plotted in Fig. 1. Studies published until June 2013 have been included in a prior pooled meta-analysis and were thus outside the scope of our search [13].

The inclusion criteria for the respective studies were as follows: (i) full-text articles in English; (ii) studies definitely reporting on patients who underwent isolated on-pump CABG; (iii) those involving patients on DAPT (irrespective of P2Y_12_ inhibitor type) with a withdrawal period of ≤7 days and (iv) those that documented at least 1 BARC-4 criterion. We excluded studies in which the timing of surgery was based on preoperative platelet function.

### Data collection and individual patient data integrity

All authors were asked to provide a standardized selection of parameters (see Supplementary Material, Table S2) from their original datasets in a pseudonymized fashion that prevented the identification of individual identities via a secure server in the Medical University of Graz. This uploading process was encrypted. The stored data were protected through access authorization. The received data were reviewed for completeness and accuracy [12] and checked for plausibility by comparing summary measures of individual patient data with published data and by checking plausibility of the individual values in a clinical context. Any implausibility was resolved by querying the original authors. Individual datasets were preprocessed and merged into a single data file for analysis.

### Participants

We included adult patients of any age and sex who underwent on-pump CABG during DAPT with aspirin and a P2Y_12_ receptor inhibitor. After receiving individual patient data from the identified studies, we excluded single patients undergoing off-pump CABG or combined cardiac surgery.

### Outcomes

The primary outcome was BARC-4 bleeding, which is defined as any of the following: perioperative intracranial bleeding, reoperation for bleeding, transfusion of ≥5 units of red blood cells, chest tube output of ≥2 l [8] (Supplementary Material, Table S5).

The secondary outcomes were 30-day mortality and postoperative ischaemic end-points defined according to the Academic Research Consortium-2 Consensus Document, which comprises a composite of death, myocardial infarction and stent thrombosis [9, 12].

### Risk of bias

Two authors (IL and MS) independently assessed the risk of bias for each study. Possible disagreements were resolved through consensus or consultation with a third party (EM).

The quality of each observational study was assessed using the Robins-I Tool as suggested by the Cochrane Handbook for Systematic Reviews of Interventions [14, 15].

The risk of availability bias was assessed by comparing the characteristics of studies that did and did not provide data.

### Statistical analysis

Categorical parameters were summarized using absolute and relative frequencies, whereas continuous parameters were presented as median and interquartile range (quartiles 1–3).

Incidence of BARC-4 bleeding was determined from the original datasets whenever possible. The primary analysis comparing patients with guideline-conforming P2Y_12_ receptor inhibitor withdrawal versus those with a shorter interval (reference group) was performed as a 2-stage IPD-MA. The included studies were separately analysed according to each type of P2Y_12_ inhibitor. Owing to the low number of patients, the results for prasugrel were limited to descriptive analysis, whereas those for clopidogrel and ticagrelor were analysed more comprehensively. One study that included only patients with non-guideline-conforming withdrawal and had unavailable data on the exact withdrawal time could not be included in the inferential analyses [16].

For the unadjusted comparison of guideline-conforming drug withdrawal, we used the Mantel-Haenszel method for the fixed effects model and the inverse variance method with the restricted maximum likelihood estimator of τ^2^ for the random effects model. In studies with zero cell frequencies, a continuity correction of 0.5 was applied. For meta-analysis of odds ratios (ORs) and incidences, the log and logit transform were used, respectively. Heterogeneity between studies was assessed using Cochran’s Q test, the I^2^ index and τ^2^. In this analysis, studies only providing aggregate data and not individual-level data on participants were included in a sensitivity analysis.

Logistic regression was used in the 1st stage of the multivariable analysis, employing Firth’s correction whenever the issue of sparse events (i.e. zero counts for factor levels) emerged. Demographic and procedural variables were considered for inclusion into the final multivariable logistic regression models if they had a *P*-value of <0.2 in univariable logistic regression analysis for at least half of the included studies. From this pool of potential confounders, variables were selected according to data availability in the single studies, multicollinearity concerns and medical relevance. Given that EuroSCORE II is a highly skewed variable that is defined by a logistic model, we used the logit transform of EuroSCORE II instead of the raw values [17].

In the 2nd stage, the resulting log ORs (i.e. regression coefficients) were pooled using multivariate random-effects meta-analytic models. To this end, we used restricted maximum likelihood estimation. Heterogeneity between studies was assessed using the multivariate Cochran’s Q test and I^2^ index.

Generally, the results of the random-effects models were presented as ORs and 95% confidence intervals (CI). For the main meta-analyses, results of the common-effects model are shown in addition [18].

The analysis for the primary end-point (i.e. BARC-4 bleeding) was repeated for 30-day mortality and the combined ischaemic end-point. For the primary and secondary analyses, no imputation of missing values was performed and no studies providing only aggregate data were included.

To explore the sources of heterogeneity, prespecified subgroup analyses with respect to patients with anaemia (World Health Organization definition: male: haemoglobin <13 g/dl, female <12 g/dl) and patients undergoing non-emergent CABG vs urgent/emergent CABG due to ACS were performed. While the subgroup analysis with respect to anaemia was conducted as a 2-stage meta-analysis, the relatively low number of patients undergoing non-emergent surgery only permitted a 1-stage logistic regression model with stratified study effects.

Prespecified sensitivity analyses were conducted to test the robustness of our findings with respect to study quality and drug-specific preoperative withdrawal periods. Given that the included studies had more missing EuroSCORE II data than anticipated, additional sensitivity analysis of the multivariable models was conducted with respect to multiple imputation of the EuroSCORE II [19]. To complement the subgroup analysis with respect to anaemia, we also conducted a sensitivity analysis that included anaemia as a factor into the multivariable model. We compared all findings of the 2-stage IPD-MA with the corresponding results of a 1-stage IPD-MA approach. All analyses were performed using the meta and mvmeta packages in R version 4.3.1 [20].

## RESULTS

From July 2013 to end of March 2024, a total of 26 observational studies (10 552 patients) evaluating the association between preoperative P2Y_12_ receptor inhibitors and CABG-related bleeding satisfied the inclusion criteria [16, 21–45]. There were no randomized controlled trials addressing our research question. Eventually, data from 7 studies (4837 patients) fulfilling the inclusion criteria were analysed, constituting 46% of available data [16, 21–26]. Double-reported patients included in a nationwide Swedish registry study and 1 single-centre study were excluded from the latter [23, 26]. Furthermore, 2 studies with aggregate data on BARC-4 bleeding in a total of 2711 patients undergoing on-pump CABG were included in a sensitivity analysis [27, 45].

Of the 4837 included patients 2398 (49.6%) were treated with clopidogrel, 2300 (47.6%) with ticagrelor and only 139 (2.9%) received preoperative prasugrel therapy. Baseline demographic and clinical variables according to type of P2Y_12_ receptor inhibitor are summarized in Table 1. EuroSCORE II was reported in 3420 patients. Data on preoperative unfractionated heparin, low molecular heparin and fondaparinux were available for 2458 patients, although information regarding the time of last administration was unavailable. While the clopidogrel and ticagrelor groups had mostly comparable baseline and clinical variables, patients on prasugrel were younger and had a lower prevalence of anaemia, higher EuroSCORE II and longer cardiopulmonary bypass time (CPB) time.

**Table 1:** Baseline demographic and clinical variables

Characteristic	Clopidogrel (*N* = 2398)	Ticagrelor (*N* = 2300)	Prasugrel (*N* = 139)
Age (years)	69 (62–75)	67 (61–74)	62 (55–70)
Male sex	1955 (82%)	1858 (81%)	113 (81%)
BMI (kg/m^2^)	26.9 (24.5–29.6)	26.9 (24.5–29.7)	27.0 (24.9–29.8)
Hb preoperative (g/dl)	13.9 (12.6–14.8)	13.8 (12.7–14.8)	14.1 (12.9–15.2)
Anaemia	517 (22%)	524 (24%)	10 (13%)
Platelets count (×10^9^/l)	225 (186–272)	237 (200–281)	218 (183–253)
Creatinine, (µmol/l)	84 (72–101)	84 (72–97)	82 (72–97)
Diabetes mellitus	700 (29%)	655 (29%)	47 (34%)
LVEF			
>50%	1406 (62%)	1386 (61%)	47 (35%)
31–50%	683 (30%)	746 (33%)	68 (51%)
21–30%	144 (6.3%)	122 (5.4%)	13 (9.7%)
≤20%	36 (1.6%)	26 (1.1%)	6 (4.5%)
EuroSCORE II	1.8 (1.1–3.4)	1.8 (1.1–3.1)	2.8 (1.6–5.1)
Urgency			
Elective	374 (16%)	163 (7.1%)	10 (7.2%)
Emergent/urgent	2022 (84%)	2132 (93%)	128 (93%)
Indication for CABG			
Stable CAD	396 (17%)	141 (6.1%)	26 (19%)
ACS	2002 (83%)	2159 (94%)	113 (81%)
UFH/LMWH/fondaparinux	841 (61%)	590 (63%)	125 (92%)
Aspirin perioperative	2099 (91%)	2133 (94%)	107 (77%)
Tranexamic acid	1142 (98%)	826 (97%)	139 (100%)
Institutional protocol for treatment of bleeding	444 (28%)	160 (9.9%)	80 (58%)
CPB time	78 (60–100)	75 (59–96)	95 (76–123)
Arterial grafts	1 (1–2)	1 (1–2)	1 (0–1)
Distal anastomoses	3 (2–4)	3 (3–4)	3 (3–4)
Days after discontinuation of P2Y_12_ receptor inhibitor	4.0 (1.0–6.0)	5.0 (2.0–7.0)	1.0 (0.0–1.0)
0	233 (11%)	244 (11%)	47 (43%)
1–2	502 (24%)	338 (15%)	43 (39%)
3–4	332 (16%)	419 (19%)	12 (11%)
≥5	1007 (49%)	1237 (55%)	8 (7.3%)
Guideline-conforming drug withdrawal	1007 (42%)	1656 (72%)	1 (0.7%)

All data are presented as median (IQR) or *n* (%).

Missing data report: *n* = 16 for BMI; *n* = 640 for Hb preoperative; *n* = 225 for anaemia; *n* = 685 for preoperative platelets; *n* = 23 for preoperative creatinine; *n* = 11 for diabetes mellitus; *n* = 154 for LVEF; *n* = 1417 for EuroSCORE II; *n* = 8 for urgency; *n* = 2379 for UFH/LMWH/fondaparinux; *n* = 130 for aspirin perioperative; *n* = 2677 for tranexamic acid; *n* = 1480 for institutional protocol for treatment of bleeding; *n* = 4 for CPB time; *n* = 3088 for arterial grafts; *n* = 741 for distal anastomoses; *n* = 415 for days after discontinuation of P2Y_12_ receptor inhibitor.

ACS: acute coronary syndrome; BMI: body mass index; CAD: coronary artery disease; CPB: cardiopulmonary bypass time; Hb: haemoglobin; LVEF: left ventricular ejection fraction.

### Incidence of BARC-4 bleeding, 30-day mortality and ischaemic outcomes

Outcome variables according to the type of P2Y_12_ receptor inhibitor and drug-specific withdrawal period (guideline conforming versus non-guideline conforming) are presented in Table 2. Since only 1 of the prasugrel patients had a guideline-conforming withdrawal time of at least 7 days, Table 2 does not distinguish prasugrel patients according to time of drug withdrawal. Among the 4812 patients analysed, 20% experienced BARC-4 bleeding. The incidence rate differed between the types of P2Y_12_ receptor inhibitor (pooled *P* < 0.001) and was highest in patients on prasugrel therapy.

**Table 2:** Outcome variables according to type of P2Y_12_ receptor inhibitor and drug-specific withdrawal time

Characteristic	Clopidogrel		Ticagrelor		Prasugrel
	<5 days (*N* = 1391)	≥5 days (*N* = 1007)	<3 days (*N* = 644)	≥3 days (*N* = 1656)	*N* = 139
BARC-4 bleeding	460 (33%)	99 (9.9%)	214 (33%)	135 (8.2%)	58 (42%)
RBC ≥5 units in 48 h	307 (22%)	77 (7.7%)	166 (27%)	83 (5.1%)	40 (29%)
24-h Chest tube drainage ≥2000 ml	118 (8.6%)	16 (1.6%)	43 (7.1%)	32 (2.0%)	21 (15%)
Reoperation	125 (9.0%)	45 (4.5%)	62 (9.6%)	68 (4.1%)	10 (7.2%)
Intracranial bleeding	1 (0.2%)	0 (0%)	0 (0%)	0 (0%)	0 (0%)
30-day mortality	41 (4.0%)	11 (1.1%)	33 (6.5%)	18 (1.1%)	6 (7.9%)
Postoperative ischaemic events	93 (8.7%)	28 (2.8%)	53 (9.1%)	46 (2.8%)	10 (9.2%)

BARC-4: Bleeding Academic Research Consortium type 4.

Data on 30-day mortality and postoperative ischaemic events, albeit heterogeneously defined, were available for 4256 and 4418 patients, respectively. The 30-day mortality rate and incidence of postoperative ischaemic events were 2.6% and 5.2%. Although included in the definition of the ischaemic end-point, none of the cases were reported to have stent thrombosis in the studies included herein.

Differences in preoperative withdrawal time are summarized in Table 1. As days off P2Y_12_ receptor inhibitors increased, BARC-4 bleeding rates evenly and gradually decreased (Supplementary Material, Fig. S1). The incidence of BARC-4 bleeding within <24 h of drug discontinuation was not significantly higher in ticagrelor-treated patients than in clopidogrel-treated patients (39.7% vs 27.9%; unadjusted OR 1.47; 95% CI 0.97–2.21; *P* = 0.066).

Among the patients who underwent surgery within <24 h of prasugrel discontinuation, 57% developed BARC-4 bleeding (Supplementary Material, Fig. S2).

### Association between guideline-conforming drug-specific preoperative discontinuation and BARC-4 bleeding, 30-day mortality and ischaemic outcomes

**Figure 2:**
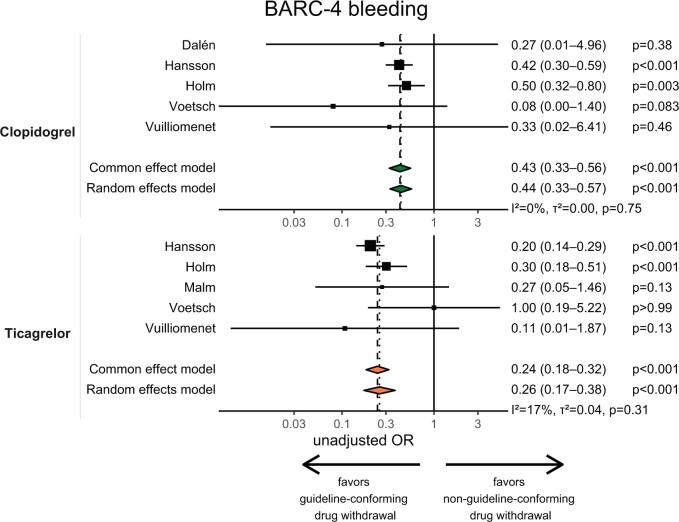
Unadjusted odds ratios for BARC-4 bleeding to compare between guideline-conforming and non-guideline-conforming preoperative discontinuation of clopidogrel and ticagrelor. Odds ratios for individual studies are represented by squares, whereas 95% confidence intervals (CI) are represented by horizontal lines. Pooled estimates and their 95% confidence intervals are represented by diamonds.

Data on BARC-4 bleeding was available for 4674 patients on clopidogrel or ticagrelor. Compared to earlier surgery, guideline-conforming discontinuation was associated with reduced risk of BARC-4 bleeding in patients on clopidogrel (unadjusted OR 0.44; 95% CI 0.33–0.57; *P* < 0.001) and ticagrelor (unadjusted OR 0.26; 95% CI 0.17–0.38; *P* < 0.001). Likewise, guideline-conforming waiting was associated with reduced risk of 30-day mortality (clopidogrel: unadjusted OR 0.42; 95% CI 0.18–0.95; *P* = 0.036; ticagrelor: unadjusted OR 0.25; 95% CI 0.13–0.47; *P* < 0.001) and reduced risk of postoperative ischaemic events (clopidogrel: unadjusted OR 0.34; 95% CI 0.22–0.52; *P* < 0.001; ticagrelor: unadjusted OR 0.34; 95% CI 0.22–0.53; *P* < 0.001) (Fig. 2 and Supplementary Material, Fig. S3). Studies evaluating BARC-4 bleeding and 30-day mortality generally had low heterogeneity.

### Multivariable analysis of BARC-4 bleeding, 30-day mortality and ischaemic outcome

**Figure 3:**
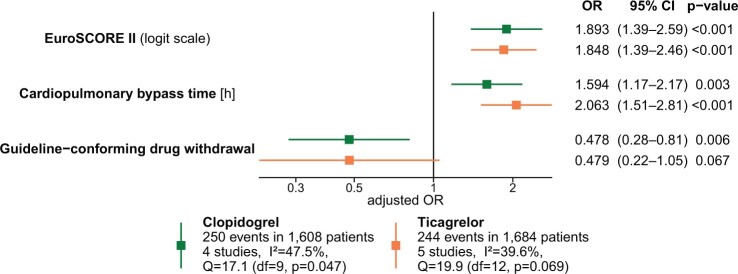
Logistic regression model for BARC-4 bleeding. Odds ratios for individual covariates are represented by squares, whereas 95% confidence intervals (CI) are represented by horizontal lines.

Patients on clopidogrel included in 1 study with systematically missing variables [21] were excluded from multivariable analysis. The contributions of covariates for BARC-4 bleeding to the multivariable model are presented in Fig. 3.

Compared to earlier surgery, guideline-conforming preoperative withdrawal time along with the logit of EuroSCORE II and CPB time was associated with decreased risk of BARC-4 bleeding in patients on clopidogrel (adjusted OR 0.48; 95% CI 0.28–0.81; *P* = 0.006) and ticagrelor (adjusted OR 0.48; 95% CI 0.22–1.05; *P* = 0.067). In the multivariable models, guideline-conforming waiting as compared to earlier surgery was significantly associated with reduced risk of postoperative ischaemic events in patients on clopidogrel (adjusted OR 0.50; 95% CI 0.30–0.82; *P* = 0.006) and not with 30-day mortality (Supplementary Material, Fig. S4).

### Association between BARC-4 bleeding and 30-day mortality

Among all analysed patients, BARC-4 bleeding was significantly associated with 30-day mortality (unadjusted OR 8.84; 95% CI 5.84–13.38; *P* < 0.001) even after adjusting for EuroSCORE II and CPB time in patients on clopidogrel and ticagrelor (adjusted OR 4.76; 95% CI 2.67–8.47; *P* < 0.001).

### Subgroup analysis

Prespecified subgroup analysis showed that the ORs for guideline-conforming preoperative withdrawal and BARC-4 bleeding did not differ significantly between anaemic and non-anaemic patients (clopidogrel P for interaction = 0.71, ticagrelor P for interaction = 0.55). Likewise, urgent or emergent surgery did not significantly influence the effect of guideline-conforming preoperative withdrawal relative to elective surgery (clopidogrel P for interaction = 0.92, ticagrelor P for interaction = 0.88) (Supplementary Material, Fig. S5).

### Sensitivity analyses

Regarding study quality, no sensitivity analysis was performed given that the 7 studies contributing to the IPD-MA were assessed to have low to moderate risk of bias comparable to the other studies (for detailed synopsis of the risk of bias, see Supplementary Material, Table S3).

Among the 19 studies not providing individual data, 2 studies presenting the respective outcome were incorporated into the sensitivity analysis. For the unadjusted primary analysis of guideline-conforming versus shorter waiting, BARC-4 bleeding and ischaemic outcomes in patients on clopidogrel, the inclusion of the aggregate data from the 2498 patients undergoing on-pump CABG reported by Qu [27] corroborated our results (unadjusted OR 0.44; 95% CI 0.35–0.56; *P* < 0.001, and unadjusted OR 0.40; 95% CI 0.27–0.61; *P* < 0.001, respectively).

In contrast, inclusion of aggregate data on BARC-4 bleeding in 213 patients undergoing CABG within 0–5 days of last ticagrelor as reported by Ingrassia [45] changed the results to unadjusted OR 0.33; 95% CI 0.19–0.57; *P* < 0.001. The sensitivity analysis revealed significant subgroup differences between the studies provided reporting individual patient data and the study of Ingrassia (*Q* = 4.04; df = 1; *P* = 0.045) [45].

Although anaemia was not included as a factor in the main multivariable model due to systematically missing data, we did include it in our sensitivity analysis. Accordingly, the inclusion of anaemia into the multivariable model for BARC-4 bleeding revealed that this factor was significant in patients on both clopidogrel (adjusted OR 1.97; 95% CI 1.23–3.17; *P* = 0.005) and ticagrelor (adjusted OR 1.85; 95% CI 1.17–2.93; *P* = 0.009), though it did not markedly change the overall results. For 30-day mortality and ischaemic outcomes, anaemia was not significant in the multivariable model (Supplementary Material, Fig. S6).

Imputation of missing EuroSCORE II values corroborated the results for the multivariable analysis, with the only difference from the main analysis being that guideline-conforming drug withdrawal remained a significant predictor for BARC-4 bleeding in patients on ticagrelor in the multivariable model (adjusted OR 0.47; 95% CI 0.24–0.91; *P* = 0.026). This suggests that the lack of significance in the main analysis might have been due to the sample size. Using univariate instead of multivariate methods in the 2nd stage of the 2-stage meta-analysis did not affect the overall results. One-stage analysis using logistic regression with stratified study effects and random effect of drug withdrawal yielded comparable results for both the unadjusted OR and multivariable model, albeit with narrower confidence intervals.

## DISCUSSION

This IPD-MA systematically reviewed and pooled studies that evaluated whether type of preoperative P2Y_12_ receptor inhibitor and preoperative withdrawal time (guideline-conforming or shorter) were associated with major CABG-associated bleeding, 30-day mortality and postoperative ischaemic events. We synthetized data from 4837 patients to assess the incidence of BARC-4 bleeding, which has been introduced as a standardized definition for bleeding among patients on antithrombotic therapy, despite the lack validation studies in cardiac surgery, for safety comparisons among studies [8]. In heterogeneous patient groups, the overall incidence rate of BARC-4 bleeding was 20%. BARC-4 bleeding gradually but variably decreased up to 5 days after drug withdrawal, which is consistent with the known recovery of platelet function [7]. Guideline-conforming preoperative withdrawal was associated with a 74% and 56% reduction in the risk of BARC-4 bleeding in patients on ticagrelor and clopidogrel as compared to a shorter preoperative withdrawal period. After incorporating EuroSCORE II, which reflects the severity of comorbidities and cardiac disease, and CPB time as markers of coagulopathic bleeding and surgical complexity in the model, guideline-conforming waiting was associated with a 52% reduction in the risk of BARC-4 bleeding in patients on ticagrelor and clopidogrel as compared to a shorter preoperative withdrawal period [17, 46]. Among patients on ticagrelor, a statistically significant reduction was only achieved after imputation of EuroSCORE II, suggesting a lack of power. Indeed, the missing EuroSCORE II values in 29% of all included patients substantially decreased the available sample size in the primary analysis.

The 2.6% 30-day mortality rate observed in this IPD-MA was comparable to the results reported by a recent post-hoc analysis of the Transfusion Avoidance Study, which evaluated 28-day in-hospital mortality risk across different Universal Definition of Perioperative Bleeding in Adult Cardiac Surgery (UDPB) and European Coronary Artery Bypass Graft bleeding severity grades (E-CABG) in patients who underwent urgent and emergent cardiac on-pump surgery [47].

Concerns have been raised regarding suboptimal antiplatelet therapy while awaiting CABG to trigger ischaemic events in patients with ACS, although reports have shown that the risk for such events is quite low [2]. The results of this IPD-MA neither support nor refute this concern considering our low event rates, missing data, heterogeneous definition of postoperative ischaemic events and wide confidence intervals. However, our findings showed that guideline-conforming withdrawal of clopidogrel reduced the risk of postoperative ischaemic events by 50% as compared to a shorter preoperative withdrawal period. Notably, we were unable to assess whether new ischaemic events occurred while awaiting surgery.

Our IPD-MA can be superior to a standard meta-analysis considering that it allows for the identification of clinical and procedural variables potentially associated with surgery-related bleeding in addition to drug-specific preoperative withdrawal time. For the current IPD-MA, we screened studies published from July 2013 to March 2024 given that those published until June 2013 had already been included in prior pooled meta-analyses [13, 48]. This IPD-MA further serves to highlight the lack of randomized controlled trials in this field.

Findings regarding increased bleeding with recent P2Y_12_ receptor inhibitor exposure reported in prior pooled IPD-MA are consistent with those presented in the current study, although prior meta-analyses had only considered reoperation for bleeding [13, 48]. While reoperation for bleeding is an integral part of the 3 consensus-based bleeding definitions, namely BARC-4, UDPB and E-CABG score, its mere consideration may underestimate severe bleeding and does not necessarily reflect coagulopathic bleeding. In fact, a recent pooled meta-analysis by Biancari *et al.* including 51 497 patients revealed that as many as two-thirds of patients undergoing re-exploration after adult cardiac surgery had surgical bleeding sites [49].

Moreover, preoperative anaemia may skew the incidence of BARC-4 bleeding given that patients with anaemia are more likely to receive red blood cell transfusions, with the amount of such transfusions constituting another major component of all 3 consensus-based bleeding definitions. Prespecified subgroup analysis demonstrated that the efficacy of guideline-conforming preoperative withdrawal of ticagrelor and clopidogrel on reducing BARC-4 bleeding was similar in patients with and without anaemia. However, the sensitivity analysis showed that anaemia nearly doubled the odds of BARC-4 bleeding.

### Clinical implications

Current guidelines have issued a class IIa recommendation for a standardized drug-specific preoperative withdrawal of P2Y_12_ receptor inhibitors in patients undergoing non-emergent cardiac surgery [1, 2, 4] and a class I indication for a preoperative discontinuation within at least 24 h in patients needing urgent CABG based on single studies [1]. The results of this IPD-MA, which synthesized data for 4837 patients from 7 observational studies, confirm and extend previous knowledge by additionally considering the impact of bleeding-relevant covariates EuroSCORE II and CPB-time thereby supporting and underscoring these ESC/EACTS and AHA/ACC recommendations. Ticagrelor, although being a more potent platelet inhibitor than clopidogrel, has been shown to wear off more quickly than clopidogrel [50]. This finding supports that earlier surgery (3 days post ticagrelor cessation) is safe in terms of bleeding, irrespective of EuroSCORE II and CPB-time and is consistent with the findings in this study. In contrast, the substantial 28% and 40% incidence of BARC-4 bleeding within 24 h of last clopidogrel or ticagrelor dose supports the recommendation to postpone urgent surgery for at least 1 day [1].

Platelet function testing may be considered as an alternative to guide decisions on timing of cardiac surgery in patients who have recently received P2Y_12_ inhibitors (class IIb recommendation); however, validated bleeding cutoffs have been lacking [7, 51]. Ongoing studies are investigating drug absorption and reversal of ticagrelor, which may eventually change the management of cardiac surgery patients on P2Y_12_ inhibitors; nonetheless, conclusive studies are still pending [52, 53].

### Limitations

The current IPD-MA only included observational studies from Europe. In the predefined search period from July 2013 to March 2024, only 1 RCT had been identified [12, 54]. In this study, however, the timing of surgery was based on the measured platelet inhibition. The use of IPD-MA does not overcome the limitations inherent to non-randomized studies, including treatment allocation bias, as well as substantial heterogeneity in outcome definitions and data availability and data quality. There is substantial centre-specific variation in the management of patients presenting for CABG while on dual antiplatelet therapy [5]. It is recommended (class Ic) that a heart team estimates the individual bleeding and ischaemic risks to time surgery and therefore it is conceivable that a primarily intended earlier surgery in unstable patients was suspended in favour of a recommended drug-specific discontinuation period [51]. Ischaemic events in the available 7 studies were heterogeneously defined and whether guideline conforming waiting as compared to earlier surgery was associated with new preoperative ischaemic events cannot be answered from this IPD-MA. However, BARC-4 bleeding was calculated according to the individually provided BARC-4 criteria.

Despite repeated approaches, datasets for only 7 out of 26 reviewed studies were received, constituting 4837 of 10 552 on-pump patients reported in the respective studies, a finding consistent with other IPD-MAs [55]. Although we did not obtain individual participant data for 19 of the studies identified, the risk of availability bias remains low for the following reasons. First, the majority of these studies (11 studies) had a small sample size of <150 patients and/or had no information regarding the incidence of BARC-4 bleeding in the original paper (14 studies). Second, only 9 studies included patients who discontinued their P2Y_12_ receptor inhibitor according to the guidelines, as well as those who did not comply with the guidelines. Among the 19 studies, only 2 were assessed to have moderate risk of bias, with the rest having moderate to severe or severe risk of bias (Supplementary Material, Table S4). Aggregated data of the largest study not included in this IPD-MA demonstrated a similar reduction in BARC-4 bleeding with guideline-conforming waiting in the 2498 patients on clopidogrel undergoing on-pump surgery [27]. In contrast, aggregated data of 213 patients on ticagrelor did not demonstrate a significant reduction of BARC bleeding depending on duration of ticagrelor withdrawal, albeit with wide confidence intervals [45]. Importantly, retrieval of the aforementioned unavailable studies would have only marginally increased the number of patients on prasugrel.

Owing to missing data in 49% of the patients and unknown last exposure of low molecular weight heparin, unfractionated heparin and fondaparinux, this IPD-MA could not determine whether preoperative anticoagulant agents had a potential additional influence on the incidence of BARC-4 bleeding. In the multicentre E-CABG registry, preoperative exposure to low molecular weight heparin, unfractionated heparin and fondaparinux was not associated with an increased risk of resternotomy after adjusting for multiple covariates and participating centres [5]. However, these drugs significantly increased the risk of severe or massive grade 2–3 E-CABG bleeding and grade 3–4 UDPB bleeding by 34% and 50%, respectively [5].

## CONCLUSION

Within the aforementioned limitations due to the lack of RCTs, the current IPD-MA supports and underscores recent guidelines by demonstrating that standardized preoperative withdrawal of ticagrelor and clopidogrel was associated with a 50% reduction in the risk of BARC-4 bleeding after correcting for EuroSCORE II and cardiopulmonary bypass time. Moreover, guideline-conforming preoperative drug discontinuation was not associated with an increased risk of 30-day mortality or postoperative ischaemic events.

Reduction of bleeding risk without concurrently demonstrating increased risk of ischaemic events or mortality may reinforce a broader adherence to guideline-conforming drug-specific preoperative withdrawal of P2Y_12_ receptor inhibitors and improve outcome after CABG.

## Supplementary Material

ezae265_Supplementary_Data

## Data Availability

The data underlying this article were provided by the authors of the original studies with their permission. Data will be shared on request to the corresponding author with permission of the authors of the original studies.

## ABBREVIATIONS

ACC: American College of Cardiology
AHA: American Heart Association
BARC: Bleeding Academic Research Consortium
CABG: Coronary artery bypass grafting
CI: Confidence interval
CPB: Cardiopulmonary bypass time
DAPT: Dual antiplatelet therapy
EACTS: European Association for Cardio-Thoracic Surgery
E-CABG: European Coronary Artery Bypass Graft bleeding severity
ESC: European Society of Cardiology
IPD-MA: Individual patient data meta-analysis
OR: Odds ratio
RCT: Randomized controlled trial
