# Supplementary material for: Platelet inhibitor withdrawal and outcomes after coronary artery surgery: an individual patient data meta-analysis
Source: Eur J Cardiothorac Surg. 2024 Jul 5;66(1):ezae265. doi: 10.1093/ejcts/ezae265 (PMC11246165; doi:10.1093/ejcts/ezae265)
Supplement: ezae265_Supplementary_Data [file ezae265_supplementary_data.zip › SupplementalTables_final_24_05_2024.docx]

## Supplemental Table 1: Search strategies for PubMed and Embase

| **Search strategy for PubMed (July 2013 – March 2024)** | |
| --- | --- |
| **ID** | **Query** |
| #1 | (clopidogrel[Title/Abstract] OR Prasugrel[Title/Abstract] OR Ticagrelor[Title/Abstract] OR dual antiplatelet[Title/Abstract] OR dual-antiplatelet [Title/Abstract] OR P2Y12 receptor inhibitor[Title/Abstract] OR p2y12 receptor antagonist[Title/Abstract] OR p2y12 Inhibitor[Title/Abstract] OR p2y12-inhibitor[Title/Abstract] OR P2Y12 inhibit*[Title/Abstract] OR P2Y12-Inhibit*[Title/Abstract] OR platelet aggregation inhibitor*[Title/Abstract] OR thienopyridine[Title/Abstract] OR ADP receptor blocking agent[Title/Abstract] OR ADP receptor antagonist[Title/Abstract] OR ADP-receptor antagonist[Title/Abstract] OR Adenosine diphosphate receptor antagonist[Title/Abstract] OR ADP-Receptor*[Title/Abstract] OR ADP Receptor*[Title/Abstract]) |
| #2 | Purinergic p2y receptor antagonists[MeSH] |
| #3 | #1 OR #2 |
| #4 | cabg[Title/Abstract] OR cardiac surgery[Title/Abstract] OR coronary artery bypass[Title/Abstract] OR Coronary artery surgery[Title/Abstract] OR coronary surgery[Title/Abstract] OR Coronary bypass surgery[Title/Abstract] OR heart surgery[Title/Abstract] OR ""on pump""[Title/Abstract] OR on-pump[Title/Abstract] OR Coronary revascularization[Title/Abstract] OR Coronary revascularisation[Title/Abstract] OR myocardial revascularization[Title/Abstract] OR myocardial revascularisation[Title/Abstract] OR ((coronary[Title/Abstract] OR cardiac[Title/Abstract]) AND (bypass[Title/Abstract] OR surgery[Title/Abstract] OR surgical[Title/Abstract] OR operation[Title/Abstract] OR operative[Title/Abstract])) |
| #5 | Coronary Artery Bypass[MeSH] OR myocardial revascularization[MeSH] |
| #6 | #4 OR #5 |
| #7 | bleeding[Title/Abstract] OR bleed*[Title/Abstract] OR hemorrhage[Title/Abstract] OR hemorrhag*[Title/Abstract] OR haemorrhage[Title/Abstract] OR haemorrhag*[Title/Abstract] OR barc[Title/Abstract] OR Blood loss[Title/Abstract] |
| #8 | Blood Loss, Surgical[MeSH] OR hemorrhage[MeSH Terms] |
| #9 | #7 OR #8 |
| #10 | #3 AND #6 AND #9 |
| #11 | **2013/07/01"[Date - Publication] : "3000"[Date - Publication]** |
| #12 | **#10 AND #11** |
| #13 | #12 AND **english[Language]** |
| **Search strategy for Embase (January 2014 – March 2024)** | |
| **ID** | **Query** |
| #1 | (clopidogrel OR Prasugrel OR Ticagrelor OR dual antiplatelet* OR dual-antiplatelet* OR P2Y12 receptor inhibitor OR p2y12 receptor antagonist OR p2y12 Inhibitor OR p2y12-inhibitor OR P2Y12 inhibit* OR P2Y12-Inhibit* OR platelet aggregation inhibitor* OR thienopyridine* OR ADP receptor blocking agent* OR ADP receptor antagonist* OR ADP-receptor antagonist* OR Adenosine diphosphate receptor antagonist OR ADP-Receptor* OR ADP Receptor*)ab,ti |
| #2 | (cabg OR cardia* surg* OR coronary artery bypass OR Coronary artery surg* OR coronar* surg* OR Coronary bypass surg* OR heart surg* OR on pump OR on-pump OR Coronar* revasculari* OR myocardial revasculari*)ab,ti |
| #3 | (bleed* OR hemorrhag* OR haemorrhag* OR barc OR Blood los*)ab,ti |
| #4 | (Blood adj3 los)ab,ti |
| #5 | #3 OR #4 |
| #6 | #1 AND #2 AND #5 |
| #7 | #6 AND English language |
| #8 | #7 AND 2014 - current |

## Supplemental Table 2: Parameters requested from individual studies (adopted from [12])

|  | Parameter | Categories/Unit |  |
| --- | --- | --- | --- |
| demographics | Age^1^ | years |  |
|  | Gender^1^ | m/f |  |
|  | Weight | kg |  |
|  | Height | m |  |
|  | BMI | kg/m² |  |
|  | Creatinine | mg/dl or µmol/l |  |
|  | Creatinine clearance^1^ | ml/min |  |
|  | Diabetes mellitus | y/n |  |
|  | Liver disease | y/n |  |
|  | LVEF^1^ | % |  |
|  | EuroSCORE II |  |  |
|  | UFH or LMWH or fondaparinux (within 24 h preop) | y/n |  |
| Procedural variables | Urgency^1^ | Elective/urgent/emergency/salvage |  |
|  | CABG indication | Stable CAD/NSTEMI/STEMI |  |
|  | CPB time | minutes |  |
|  | Number of arterial grafts |  |  |
|  | Number of distal anastomoses |  |  |
|  | Tranexamic acid during surgery | y/n |  |
|  | Hemoglobin preoperative | g/l |  |
|  | Platelets preoperative | x10^9^/l |  |
|  | Institutional protocol for treating post-pump bleeding | y/n |  |
|  | Aspirin perioperative continuation | y/n |  |
|  | Aspirin cessation prior to surgery | days |  |
|  | Clopidogrel preoperative | y/n |  |
|  | Clopidogrel cessation prior to surgery | days |  |
|  | Prasugrel preoperative | y/n |  |
|  | Prasugrel cessation prior to surgery | days |  |
|  | Ticagrelor preoperative | y/n |  |
|  | Ticagrelor cessation prior to surgery | days |  |
| outcome | Chest tube drainage volume within 24 h^2^ | ml |  |
|  | Reoperation due to bleeding | y/n |  |
|  | Intracranial bleeding within 48 h hours perioperatively | y/n |  |
|  | Number of transfused red blood cell units within 48 h from incision |  |  |
|  | Postoperative myocardial infarction | y/n |  |
|  | In hospital mortality | y/n |  |
|  | 30-day mortality | y/n |  |

*Calculated parameter, ^1^part of EuroSCORE II, ²if unavailable chest tube drainage volume obtained during shorter observation period (define observation period)

## Supplemental Table 3: Risk of bias assessment using the Robins-I Tool

|  | **Dalen**  **[21]** | **Vuillio- menet**  **[25]** | **Voetsch**  **[24]** | **Malm**  **[26]** | **Holm**  **[22]** | **Hansson**  **[23]** | **Schlachten-berger**  **[16]** | **Qu**  **[27]** | **Ingrassia [45]** |
| --- | --- | --- | --- | --- | --- | --- | --- | --- | --- |
| **Bias due to confounding** | moderate | moderate | moderate | moderate | moderate | low | low | low | Moderate |
| **Bias in selection of participants into the study** | low | moderate | low | low | moderate | low | low | low | low |
| **Bias in classification of interventions** | moderate | low | low | moderate | low | low | moderate | low | low |
| **Bias due to deviations from intended interventions** | low | low | low | low | low | low | low | low | low |
| **Bias due to missing data** | low | moderate | low | low | low | low | low | low | low |
| **Bias in measurement of outcomes** | moderate | moderate | low | moderate | low | low | low | moderate | low |
| **Bias in selection of the reported result** | low | low | low | low | low | low | low | low | low |
| **Overall bias** | moderate | moderate | low/moderate | moderate | moderate | low | low/moderate | low/moderate | low/moderate |

## Supplemental Table 4: Study characteristics

| First author | Country | Data received | Design | Multi-center | BARC-4 | ROB | Off-pump included | Combination surgery included | Clopidogrel | Ticagrelor | Prasugrel | Acc. to guideline | Not acc. to guideline | n |
| --- | --- | --- | --- | --- | --- | --- | --- | --- | --- | --- | --- | --- | --- | --- |
| Dalén M  [21] | Sweden | y | prospective observational | n | n | moderate | n | n | y | y | n | y | y | 86 |
| Holm M  [22] | multiple | y | prospective observational | y | y | moderate | n | n | y | y | n | y | y | 1,480 |
| Hansson EC  [23] | Sweden | y | retrospective observational | y | y | low | n | n | y | y | n | y | y | 2,321 |
| Vuilliomenet T  [25] | Switzer-land | y | retrospective observational | n | y | moderate | n | n | y | y | y | y | y | 223 |
| Voetsch A  [24] | Austria | y | retrospective observational | n | y | low-moderate | n | n | y | y | y | y | y | 269 |
| Schlachtenberger G [16] | Germany | y | retrospective observational | n | y | low-moderate | n | n | y | y | y | n | y | 415 |
| Malm CJ  [26] | Sweden | y | prospective observational | n | y | moderate | n | n | n | y | n | y | y | 88 |
| Qu  [27] | China | n | retrospective observational | n | y | moderate | y | n | y | n | n | y | y | 2,498 |
| Ingrassia JJ  (45) | USA | n | Retrospective observational | n | y | low-moderate | n | n | y | y | n | y | y | 213 |
| Petricevic M  [28] | Croatia | n | retrospective observational | n | n | moderate-severe | ? | n | y | n | n | y | y | 277 |
| Diab S  [29] | Israel | n | retrospective observational | n | n | moderate-severe | n | y | n | y | n | n | y | 53 |
| Tomšič A  [30] | Nether-lands | n | retrospective observational | n | n | moderate | n | n | y | y | n | y | y | 222 |
| Charif F  [31] | Lebanon | n | retrospective observational | n | n | severe | n | n | y | n | n | n | y | 126 |
| Nagashima Z  [32] | Japan | n | retrospective observational | n | y | severe | n | n | y | n | n | n | y | 15 |
| Russo JJ  [33] | Canada | n | retrospective observational | n | y | moderate-severe | ? | n | y | y | n | y | y | 508 |
| Yu PJ  [34] | USA | n | prospective observational | n | n | moderate-severe | ? | n | y | n | n | y | y | 71 |
| Schotola H  [35] | Germany | n | retrospective observational | n | n | moderate-severe | n | n | y | y | n | n | y | 68 |
| Drews S  [36] | Switzer-land | n | retrospective observational | n | n | moderate-severe | n | n | y | n | y | y | y | 143 |
| Schaefer A  [37] | Germany | n | retrospective observational | n | n | severe | y | y | y | y | n | n | y | 20 |
| Amour J  [38] | France | n | retrospective observational | n | n | severe | n | n | y | n | n | n | y | 61 |
| Kremke M  [39] | Denmark | n | retrospective observational | n | n | severe | y | y | n | y | n | y | y | 90 |
| Nardi P  [40] | Italy | n | retrospective observational | n | n | severe | y | n | y | y | n | y | y | 307 |
| Seese L  [41] | USA | n | retrospective observational | n | n | severe | y | n | y | n | n | n | y | 800 |
| Woźniak S  [42] | Poland | n | prospective observational | n | y | severe | y | n | y | n | n | ? | ? | 24 |
| Plicner D  [43] | Poland | n | Case-control | n | n | moderate-severe | n | n | y | n | n | n | y | 52 |
| Kacar S  [44] | Serbia | n | Retrospective observational | n | n | moderate-severe | n | n | y | n | n | y | y | 122 |

Studies highlighted in dark gray: included in IPD-MA;
highlighted in light gray: included in sensitivity analysis as aggregated data.

## Supplemental Table 5: Individual participant data provided by individual studies

| **Characteristic** | **Dalén [21]** | **Hansson [23]** | **Holm [22]** | **Malm [26]** | **Schlachten-berger [16]** | **Voetsch [24]** | **Vuilliomenet [25]** |
| --- | --- | --- | --- | --- | --- | --- | --- |
| **EuroSCORE II** | n | y | y | y | n | y | y |
| **Days after discontinuation  of P2Y_12_ receptor inhibitor** | y | y | y | y | n | y | y |
| **BARC-4 bleeding (combined endpoint) *** | n | y | n | y | y | y | n |
| **Single BARC-4 criteria**** |  |  |  |  |  |  |  |
| **RBC ≥ 5 units in 48h** | y | y | y | n | y | y | y |
| **24-h Chest tube drainage ≥ 2000 mL** | y | y | y | n | y | y | y |
| **Reoperation** | y | y | y | y | y | y | y |
| **Intracranial bleeding** | y | n | y | y | n | y | y |
| **30-day mortality** | y | y | y | y | n | y | y |
| **Postoperative ischemic events** | y | y | y | y | n | y | y |
| **Postoperative MI***** | y | n | y | n | n | y | y |

BARC-4 bleeding occurrence was either identified by the combined endpoint* or by at least one single BARC-4 criteria** as provided by the individual authors.

Postoperative myocardial infarction*** was heterogeneously defined throughout the seven studies.
