# Supplementary material for: Platelet inhibitor withdrawal and outcomes after coronary artery surgery: an individual patient data meta-analysis
Source: Eur J Cardiothorac Surg. 2024 Jul 5;66(1):ezae265. doi: 10.1093/ejcts/ezae265 (PMC11246165; doi:10.1093/ejcts/ezae265)
Supplement: ezae265_Supplementary_Data [file ezae265_supplementary_data.zip › SupplementalFigures_2024-06-18_final.pdf]

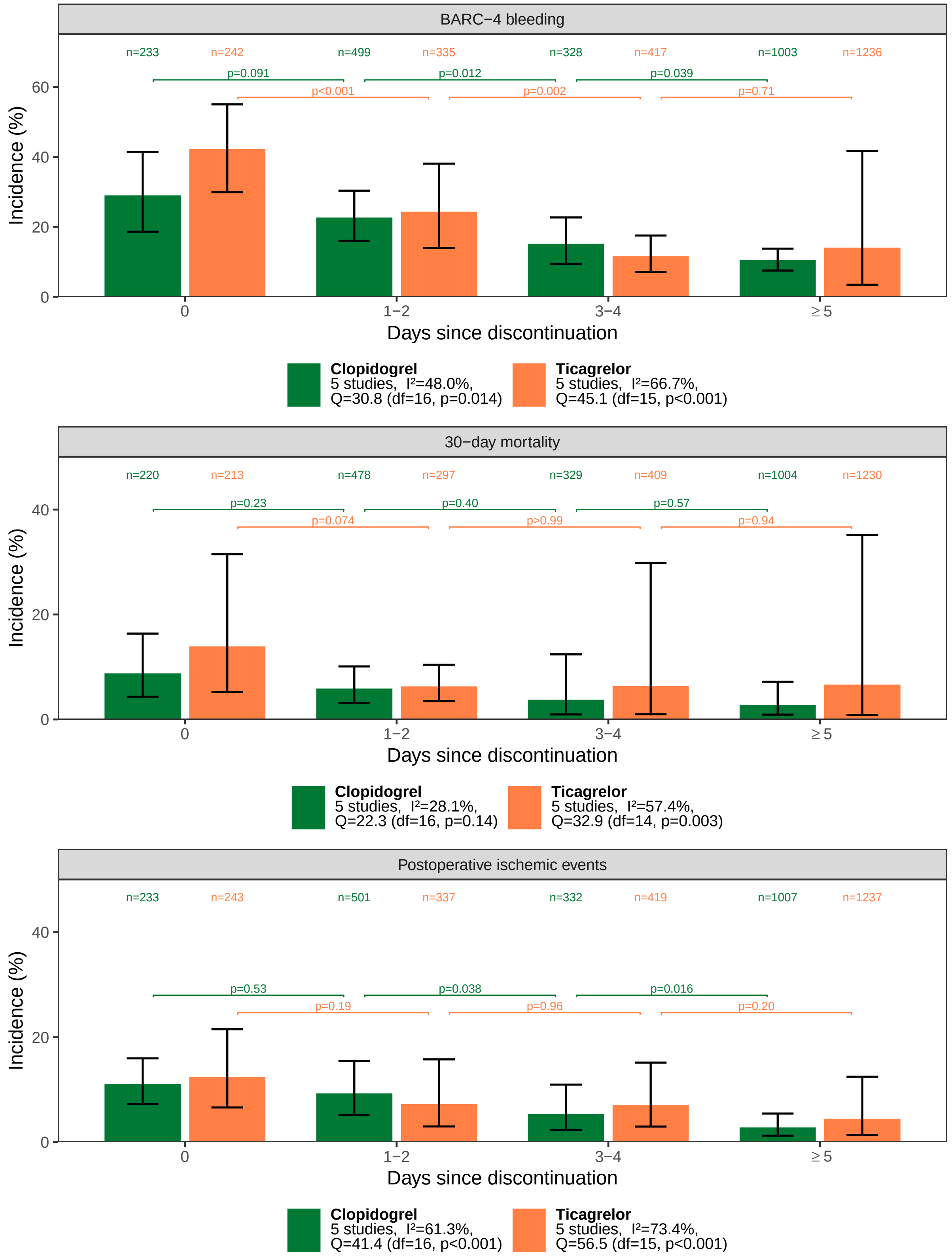

Supplemental Figure 1: Incidence of BARC-4 bleeding, 30-day mortality, and postoperative ischemic events according to type of $P2Y_{12}$ receptor inhibitor and preoperative withdrawal time.

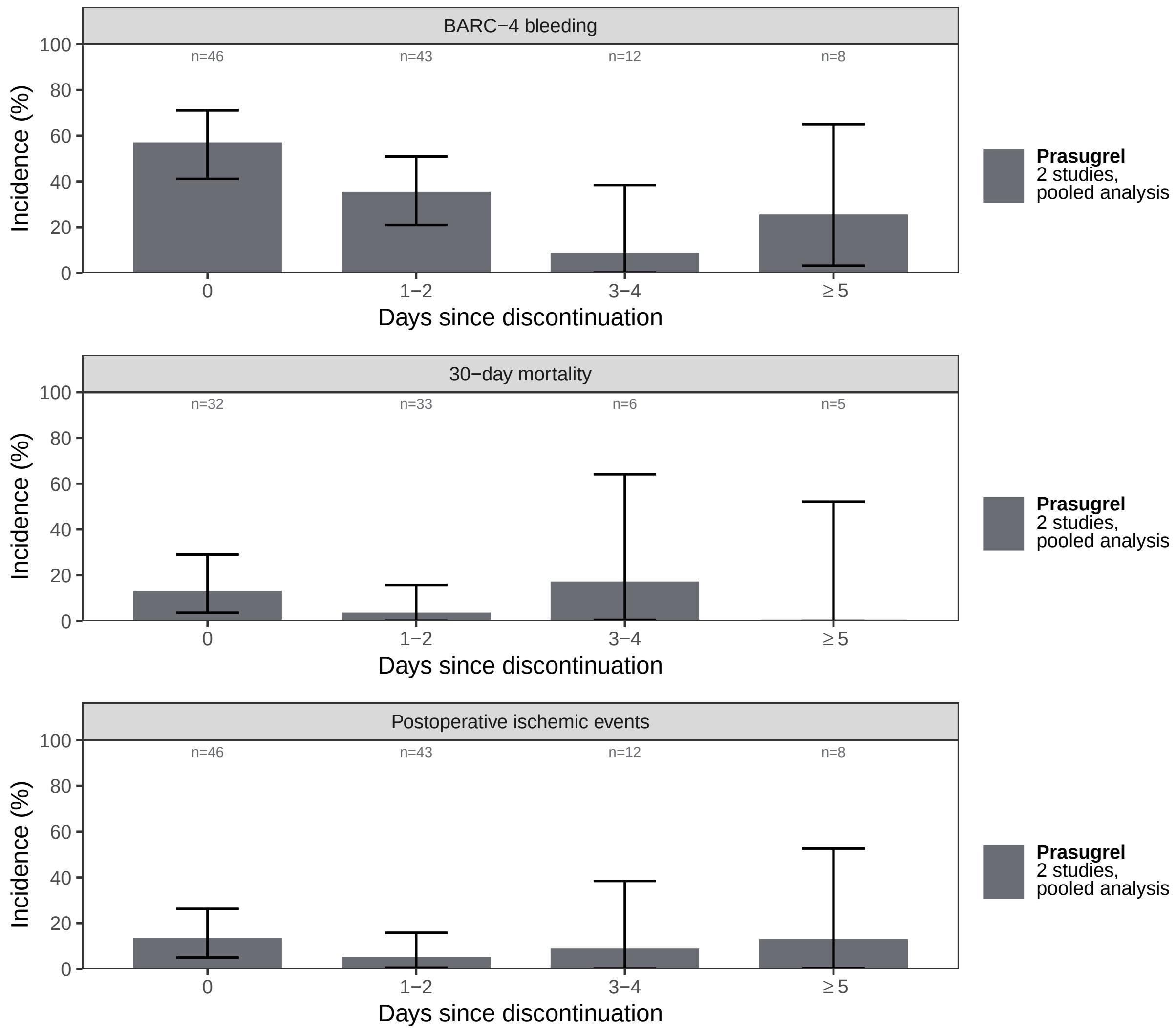

Supplemental Figure 2: Incidence of BARC-4 bleeding, 30-day mortality, and postoperative ischemic events in patients on prasugrel stratified according to preoperative withdrawal time pooled from two studies.

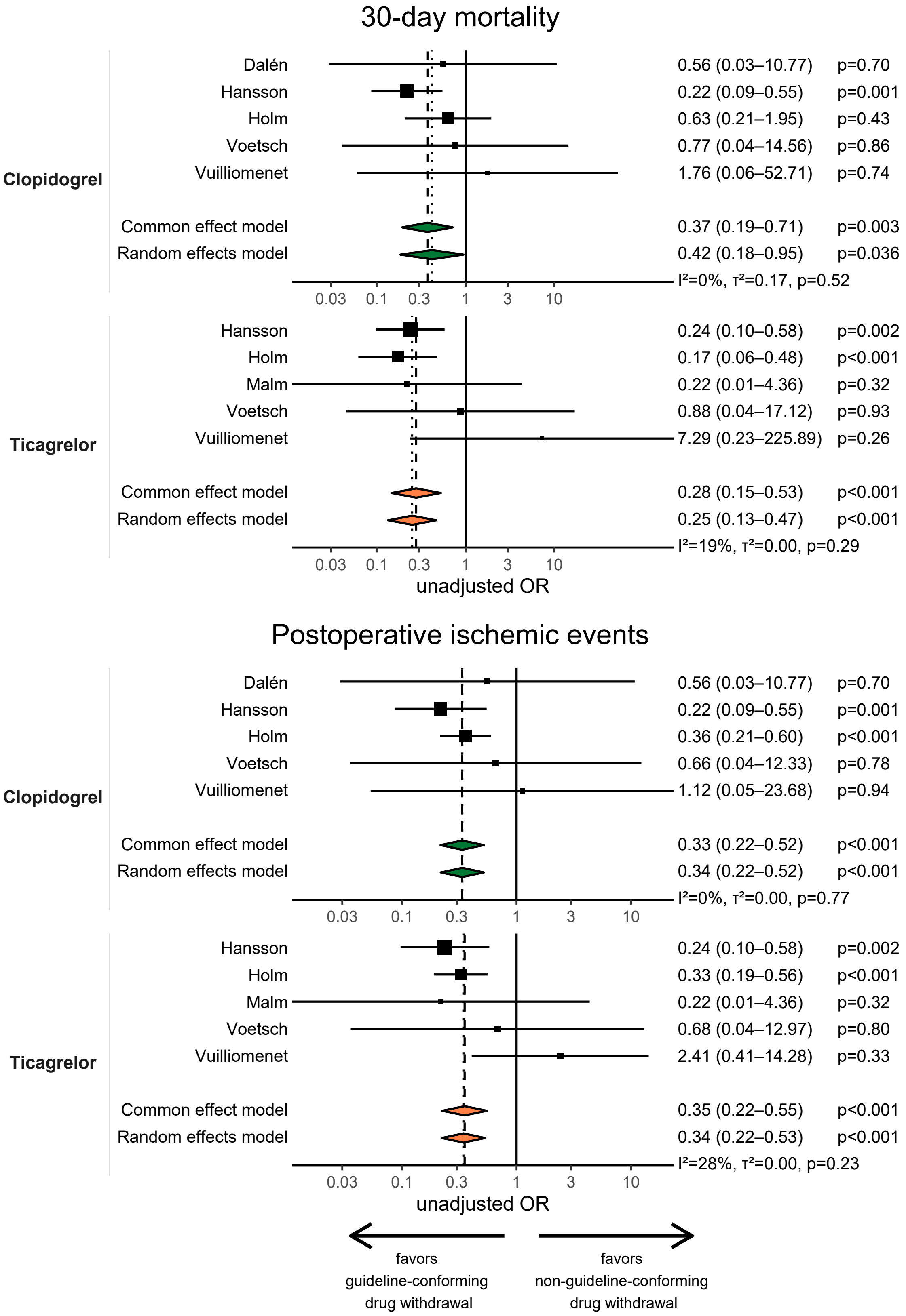

Supplemental Figure 3: Unadjusted odds ratios for 30-day mortality and postoperative ischemic events to compare between guideline-conforming and non-guideline-conforming preoperative discontinuation of clopidogrel and ticagrelor. Odds ratios for individual studies are represented by squares, whereas 95% confidence intervals (CI) are represented by horizontal lines. Pooled estimates and their 95% confidence intervals are represented by diamonds.

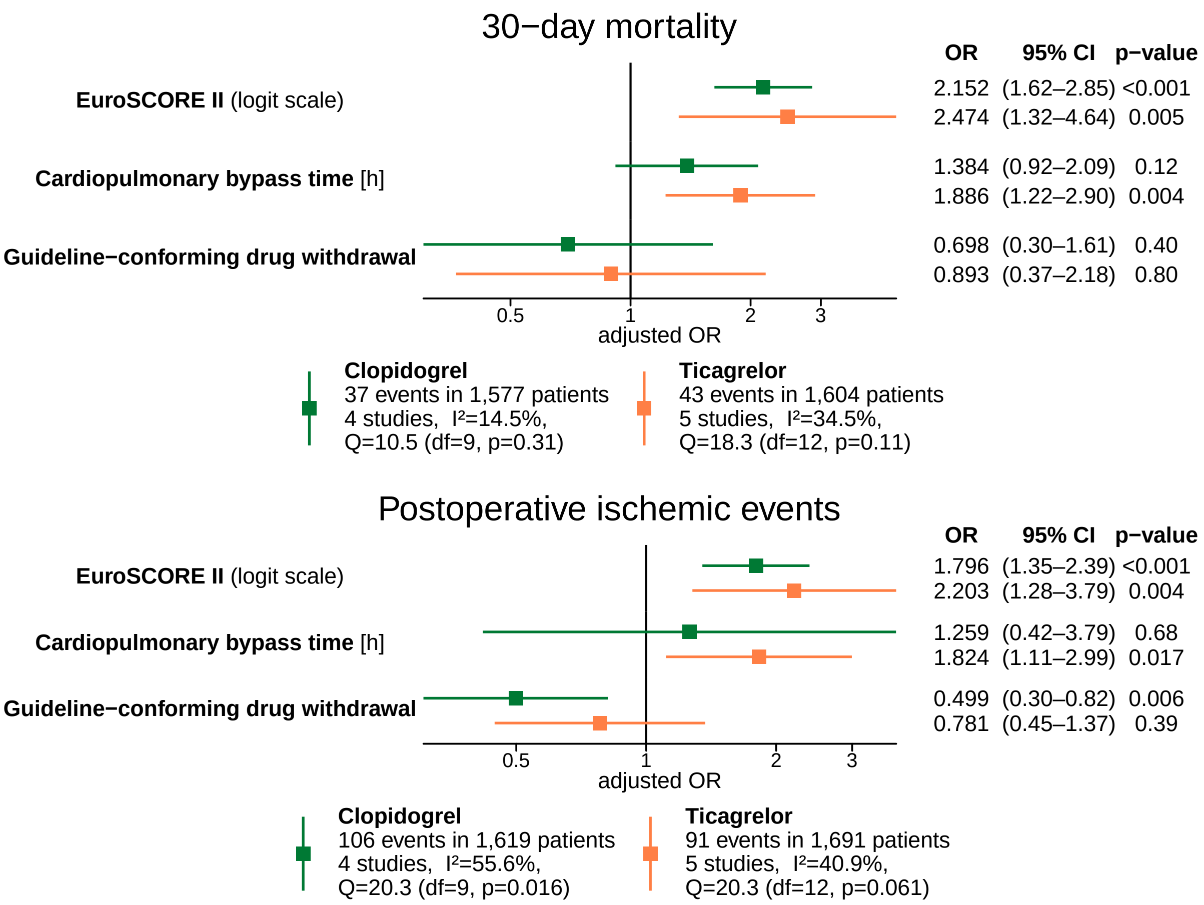

Supplemental Figure 4: Logistic regression model for 30-day mortality and postoperative ischemic events. Odds ratios for individual covariates are represented by squares, whereas 95% confidence intervals (CI) are represented by horizontal lines.

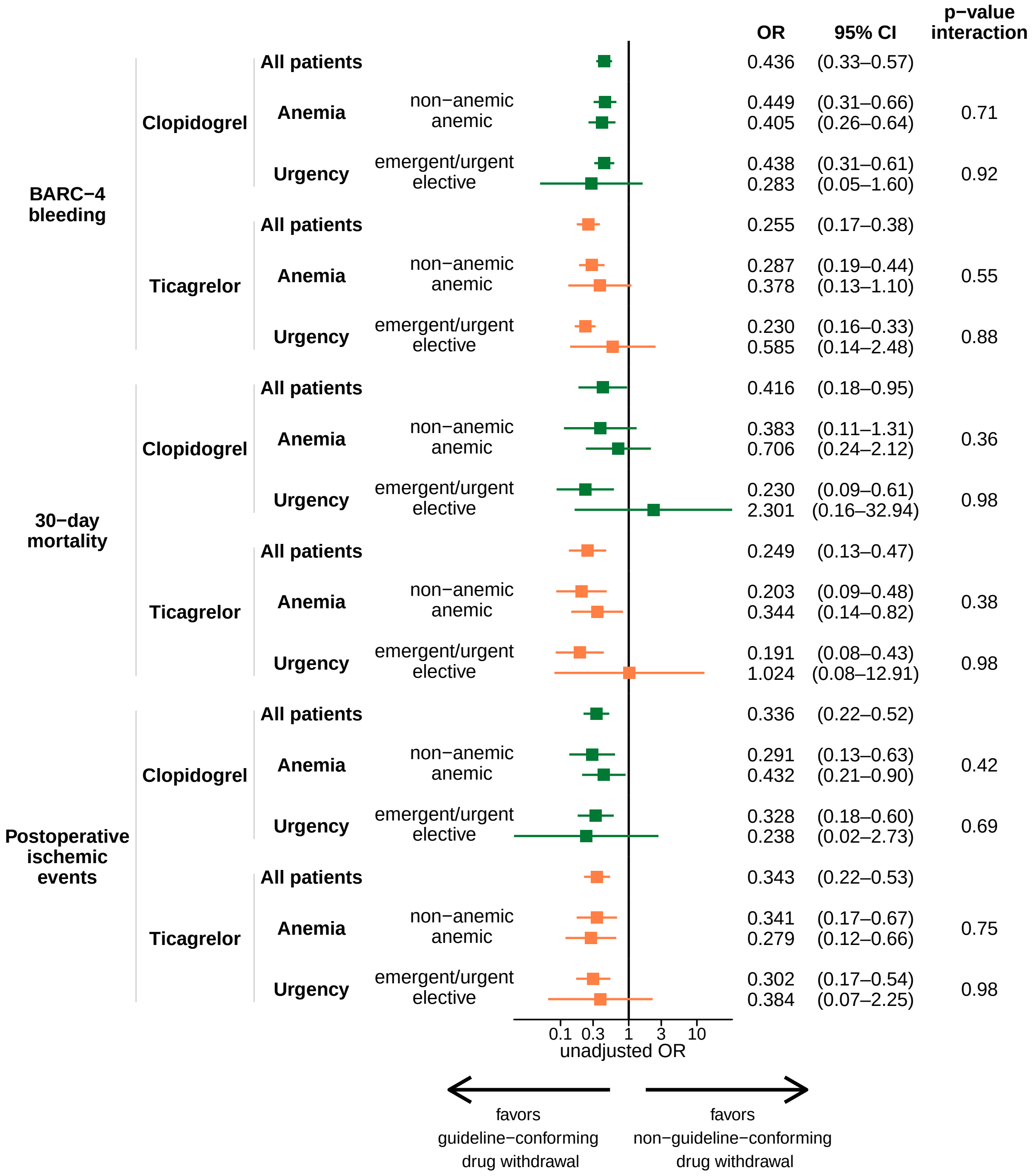

Supplemental Figure 5: Subgroup analysis on anemia and urgency of surgery in patients with acute coronary syndrome on BARC-4 bleeding, mortality, and postoperative ischemic events.

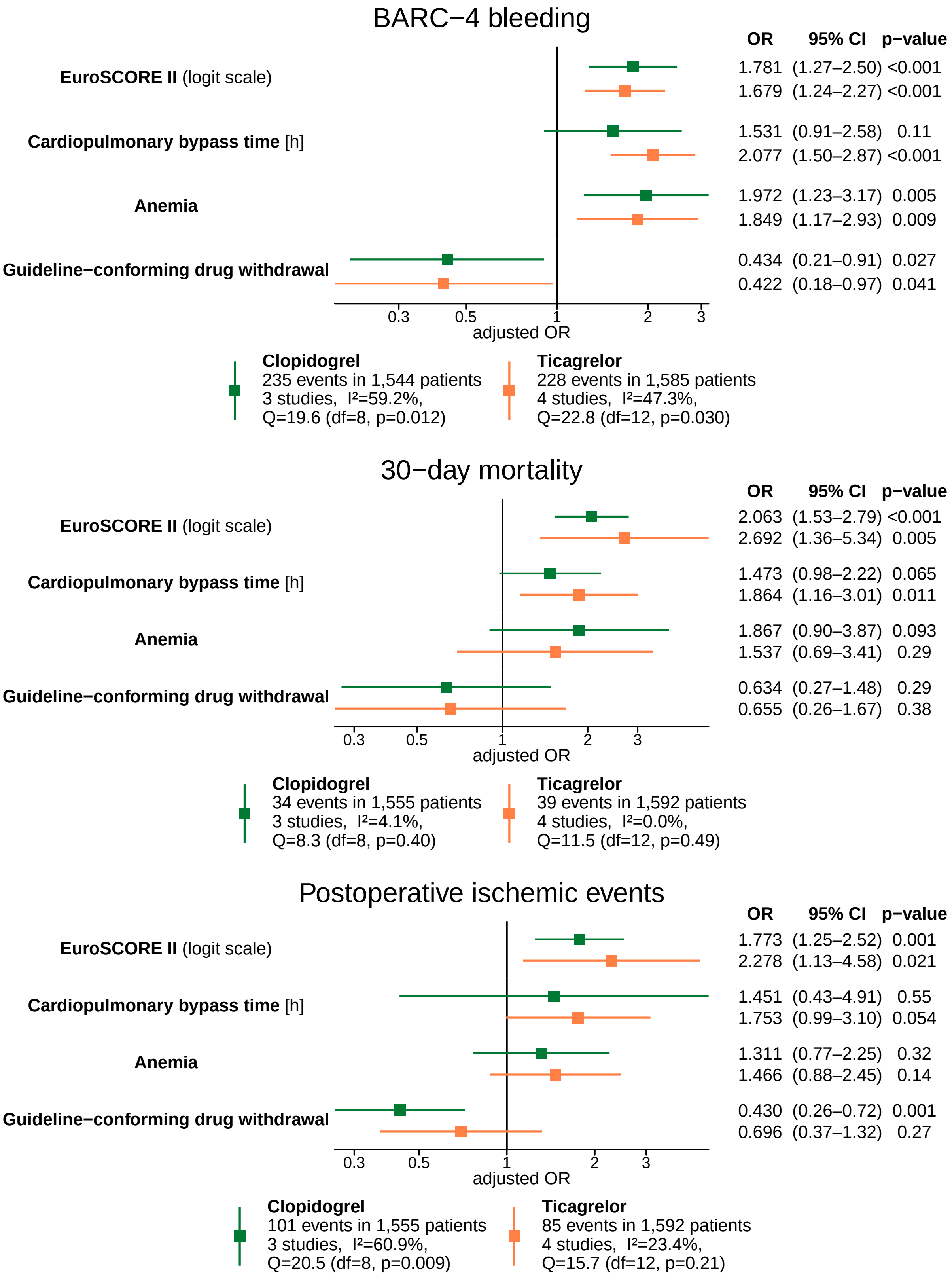

Supplemental Figure 6: Logistic regression models for BARC-4 bleeding, 30-day mortality, and postoperative ischemic events, including anemia as a covariate. Odds ratios for individual covariates are represented by squares, whereas 95% confidence intervals (CI) are represented by horizontal lines.
